# The Potential of Astrocytes as Immune Modulators in Brain Tumors

**DOI:** 10.3389/fimmu.2019.01314

**Published:** 2019-06-11

**Authors:** Neibla Priego, Manuel Valiente

**Affiliations:** Brain Metastasis Group, Molecular Oncology Programme, National Cancer Research Center (CNIO), Madrid, Spain

**Keywords:** brain tumor, brain metastasis, astrocytes, immune system, cell-to-cell communication

## Abstract

The neuro-immune axis has emerged as a key aspect to understand the normal function of the Central Nervous System (CNS) as well as the pathophysiology of many brain disorders. As such, it may represent a promising source for novel therapeutic targets. Glial cells, and in particular the extensively studied microglia, play important roles in brain disorders. Astrocytes, in their reactive state, have been shown to positively and negatively modulate the progression of multiple CNS disorders. These seemingly opposing effects, might stem from their underlying heterogeneity, an aspect that has recently come to light. In this article we will discuss the link between reactive astrocytes and the neuro-immune axis with a perspective on their potential importance in brain tumors. Based on the gained knowledge from studies in other CNS disorders, reactive astrocytes are undoubtfully emerging as a key component of the neuro-immune axis, with ability to modulate both the innate and adaptive branches of the immune system. Lastly, we will discuss how we can exploit our improved understanding of the basic biology of astrocytes to further enhance the efficacy of emerging immune-based therapies in primary brain tumors and brain metastasis.

## Astrocytes in Homeostasis and Disease

Astrocytes are involved in a variety of phisiological functions including maintenance of the blood-brain barrier (BBB) and blood flow (1), modulation of synaptic plasticity (2), and regulation of energy homeostasis (3). All of these functions have a significant impact on many aspects of our daily life such as cognition (4), fear (4), sleep (5), and circadian rhythm (6). The heterogeneity of astrocytes might contribute to these pleiotropic functions. For instance, astrocytes from the hippocampus differ functionally in multiple aspects when compared to those from the striatum (7). But even within the same brain area, astrocytes have molecular differences that functionally correlate with their ability to interact with neurons (8). Single cell-RNA sequencing (scRNAseq) will undoubtedly help to clarify not only the diversity within what we call today astrocytes as a whole but also the origin of such heterogeneity. Sources of astrocyte heterogeneity might include different progenitors during development (9) or the ability to generate new astrocytes upon injury (10, 11). Especially interesting are novel technologies that allow mapping single cell transcriptomics within tissue sections (12). Given the link between location and astrocyte function, as shown by the different biology of juxtavascular astrocytes (13), having spatial resolution of transcriptomic profiles might be key to properly interpret the many flavors of astrocytes.

In addition to their homeostatic functions in the central nervous system (CNS), astrocytes are rapidly activated in response to various insults, including brain tumors (14, 15). The activation pattern of astrocytes and its consequences appear to be dependent on the nature of the initiating pathogenic event. Moreover, this is a dynamic process that evolves throughout the course of the disease. While primarily limiting spread of the damage in the context of acute phase brain injury, astrocytes rather appear to worsen disease outcome in a chronic injury setting (16, 17). This also applies to brain metastasis, where reactive astrocytes play an anti-metastatic role that limits disease progression in early stages of brain colonization (18), while, later on, they become strongly pro-metastatic (19). Therefore, in order to fully comprehend the biological significance of astrocytes in brain physiology and pathology, we need to consider their highly plastic behavior and heterogeneous make up. These features allow astrocytes to trigger a remarkably fine-tuned response to adequately counteract a broad spectrum of injuries. Given the growing importance of the immune system and its therapeutic exploitation in many brain disorders, including cancer, addressing the biological significance of the cross-talk between immune cells and astrocytes might offer innovative means to challenge incurable CNS disorders, such as primary and secondary brain tumors.

## Influence of Astrocytes on the Innate Immune System

### Cross-Talk Between Astrocytes and Microglia

Microglia and astrocytes are resident glial cells that influence each other under homeostatic conditions (20) but also when the CNS is affected by pathology.

**Figure 1:**
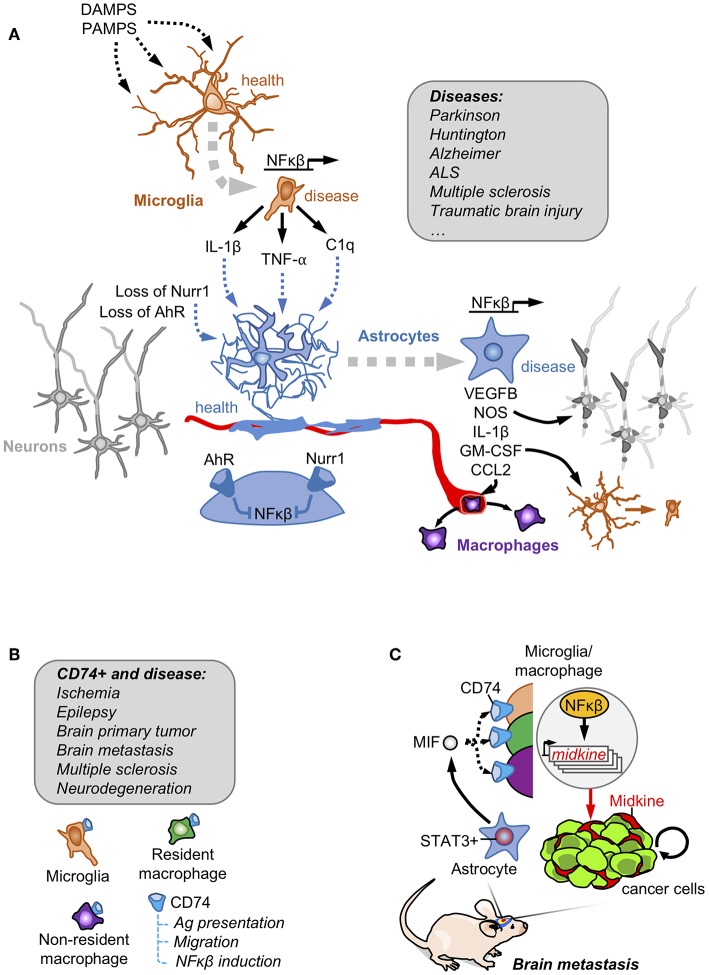
Astrocytes and innate immunity. **(A)** Danger and pathogen associated molecular patterns (DAMPS and PAMPs, respectively) are detected by microglia that become activated secreting NFκβ downstream targets. Activated microglia crosstalk with astrocytes to induce a NFκβ-dependent program responsible for damaging neuronal components, favoring the access of peripheral macrophages and reinforcing the activation of the microglia. Under basal conditions, astrocytes have this NFκβ program shut-down by the action of Nurr1 and AhR receptors. This mechanism involving the crosstalk between reactive astrocytes and microglia has been described in several disorders affecting the Central Nervous System. **(B)** Brain macrophages in disease include microglia, non-parenchymal resident macrophages and infiltrating non-resident macrophages. A subpopulation expressing the CD74 receptor has been reported to be present in all of them, especially when the brain is affected by disease. **(C)** In brain metastasis, secretion of MIF by pSTAT3+ reactive astrocytes enrich CD74+ microglia/ macrophages in the tumor where they produce the NFκβ-dependent molecule midkine that promotes tumor cell survival.

*In vitro*, the classical inducer of neuroinflammation LPS stimulates microglia to produce a secretome enriched in NFκβ-regulated molecules including IL-1, TNF and C1q. The microglia-conditioned medium was sufficient not only to turn non-activated into activated astrocytes, assessed by the gained expression of GFAP, but also to induce the production of an unidentified secreted factor/s by astrocytes that compromised the viability of neurons and oligodendrocytes (21) (Figure 1A). This particular behavior of activated astrocytes with neurotoxic properties has been suggested to be present in patients with neurodegenerative (Alzheimer, Huntington, Parkinson, amyotrophic lateral sclerosis) and autoimmune (multiple sclerosis) CNS disorders. This hypothesis was supported by the increased levels of three proteins (C3, CBF, and MX1) that were initially found to be upregulated in a transcriptional signature of microglia-stimulated astrocytes (21).

A similar crosstalk between astrocytes and microglia was probed to be involved in some rare forms of Parkinson with mutations in the orphan receptor NURR1. Under normal circumstances NURR1 blocks the activation of NFκβ-dependent genes, a function that is lost in these patients with Parkinson disease. The combination of engineered inactivating *Nurr1* mutations in glial cells with a background of increased inflammation (i.e., LPS treatment) lead to the death of dopaminergic neurons, which is a hallmark of Parkinson (22). The molecular analysis of this cross-talk probed that Nurr1-mutant astrocytes had an augmented response to microglia-derived TNFα and IL1β involving a sustained occupancy of the iNOS promoter by p65, thus secreting nitric oxide (NO) at levels that might be responsible for compromising neuronal viability (22) (Figure 1A).

The dependency of astrocyte activation on microglia behavior, was also validated *in vivo* in a mouse model of experimental autoimmune encephalomyelitis (EAE). Activation of the aryl hydrocarbon receptor (AhR) in microglia promotes the expression of TGFα. On the contrary, the absence of AhR signaling limits the expression of the NFκβ negative regulator *Socs2*, which increases the secretion of NFκβ-dependent molecules such as VEGFB (Figure 1A). Microglia secretomes enriched in either TGFα (when AhR is activated) and VEGFB (when AhR is inactivated) induced opposite transcriptomic responses when added to astrocytes by decreasing or inducing, respectively, the expression of *Ccl2, Nos2* and *IL1b*. Some of the deregulated genes were enriched in activated astrocytes with the ability to compromise the viability of neurons and oligodendrocytes (21). In fact, when *AhR* was targeted in the context of EAE, disease worsened. Furthermore, targeting *Ccl2, Nos2* and *IL1b* using cell-specific loss of function approaches either in microglia or astrocytes improved EAE outcome (23). AhR could be activated by tryptophan-derived metabolites (24). Since tryptophan is an essential amino acid provided by diet that is processed by the gut microbiome, this suggests the possibility that diet and the intestinal microbiota could have an impact on neuroinflammation. Interestingly, depleting tryptophan from the diet mimicked the phenotype of targeting *AhR* in microglia thus worsening EAE. Adding back the amino acid in the diet rescued the phenotype but only when the AhR receptor was present (23).

In summary, evidence exists about the critical influence of microglia on astrocytes in CNS disorders. The degree of activation of a NFκβ-dependent secretome in microglia defines the consequences on astrocytes. Microglia-activated astrocytes could worsen disease outcome by their negative influence on neuron and oligodendrocyte viability. Although the influence of microglia on astrocytes have been probed, whether astrocytes could influence microglia is less well-characterized (25).

### Cross-Talk Between Astrocytes and Brain-Infiltrating Monocytes

Monocytes are excluded from the healthy brain. However, when the brain gets injured, CCR2+ circulating monocytes access the parenchyma (26, 27). As a key component of the BBB, astrocytes are one of the first cell types encountered by infiltrating peripheral immune cells, which provides the glial cell a strategic position to control this transit.

Traumatic brain injury has an impact in the viability of astrocytes located in the proximity of the damaged area. Simultaneously to the decrease in astrocytes, there is an increase in the infiltration of CCR2+ monocytes, which suggests that these cell types could influence each other. Juxtavascular astrocytes are a subpopulation that interacts physically with brain vessels and proliferation upon damage (13, 28). Although this subpopulation of astrocytes has been shown to correlate with a specific developmental origin, they were not characterized at the molecular level. Recently, juxtavascular astrocytes have been shown to preferencially activate AhR. Given that AhR blocks the production of CCL2, a strong chemokine for CCR2+ monocytes, this subpopulation of astrocytes acts as a selective barrier modulating the access of peripheral cells into the brain parenchyma (28).

Monocytes also influence astrocytes. If traumatic injury is generated in a mouse without CCR2+ monocytes, higher numbers of proliferative astrocytes are detected, suggesting a deleterious influence of infiltrated monocytes on the proliferation of juxtavascular astrocytes (28). Interestingly, in spite of the increased proliferative rates of these astrocytes, the glia scar and extracellular matrix deposition surrounding the damage was reduced and consequently, better neuronal recovery was detected (28). This finding illustrates the importance of defining at the molecular level newly established cell-to-cell interactions that occur once peripheral cells from the innate immune system infiltrate the brain. It also illustrates the importance of characterizing astrocyte heterogeneity given the impact that specific astrocyte subtypes have on disease progression (28).

### Cross-Talk Between Astrocytes and Macrophages in Brain Tumors

In spite of the evidences presented in other brain pathologies, the crosstalk between astrocytes and macrophages had been barely explored in brain tumors. This is surprising given that the majority of immune cells within brain tumors are macrophages either resident or infiltrated from the periphery (27, 29, 30). Recently, astrocytes have been proved to influence a subtype of microglia/ macrophage expressing CD74.

*CD74* is among the most upregulated genes in human microglia in the context of brain tumors and other pathologies (31) (Figure 1B). The association of CD74 in microglia/macrophages and brain disorders have been recently extended and validated by scRNAseq approaches comparing healthy and brains affected by autoimmune disorders, neurodegeneration or ischemia. *Cd74* upregulation was consistently found in disease-associated macrophages including peripheral macrophages infiltrating the brain, non-parenchymal resident macrophages (meningeal, perivascular, and choroid plexus macrophages) as well as in one subclass of microglia (26, 32) (Figure 1B).

Funtionally, the CD74+ microglia/macrophages were shown to reduce the secretion of IFN-γ in the tumor microenvironment, which would contribute to established an immunosuppressed niche (33). More recently, the increase of CD74+ microglia/macrophages in the context of brain metastases was shown to be dependent on the presence of pSTAT3+ reactive astrocytes, describing a cross-talk between both cell types (19). The ligand of CD74 receptor, MIF, is highly enriched in the secretome of pSTAT3+ reactive astrocytes. CD74+ microglia/macrophages are preferentially located within the metastatic lesion. At this location CD74 could be found translocated in the nucleus where it promotes the expression of NFκβ downstream targets, such as midkine (19), a secreted molecule that accumulates in the extracellular space to promote cell viability (34) (Figure 1C). The importance of MIF binding to CD74+ microglia/macrophages was demonstrated by the reduction of brain metastasis upon treatment with the BBB-permeable MIF inhibitor ibudilast in organotypic cultures (19). Interestingly, ibudilast has been successfully used in patients with multiple sclerosis (35) and in experimental models of glioblastoma (36), which inspired a recently initiated clinical trial (NCT03782415). Although the biology of CD74+ microglia/macrophages remains poorly characterized, its strong association with different brain disorders and its diverse set of functions including the role as a chaperone for the MHCII complex (37), the modulation of migration by interacting with myosin (38) and the activation of NFκβ pathway (34) suggest relevant implications in disease.

## Influence of Astrocytes on the Acquired Immune System

In contrast to the long-term dogma that defined the brain as an immune-privileged organ, the presence of primary or secondary brain tumors correlates with a significant infiltration of CD8+ and CD4+ T cells (39–41). Given that brain infiltrating T cells and reactive astrocytes co-exist in the same spatial location surrounding the tumor (19) and the strong secretory nature of astrocytes, it is quite likely that both cell types could influence each other. The molecular regulation of this cross-talk and its consequences are emerging linked to several brain disorders including cancer.

### Cross-Talk Between T Regulatory Cells and Astrocytes

**Figure 2:**
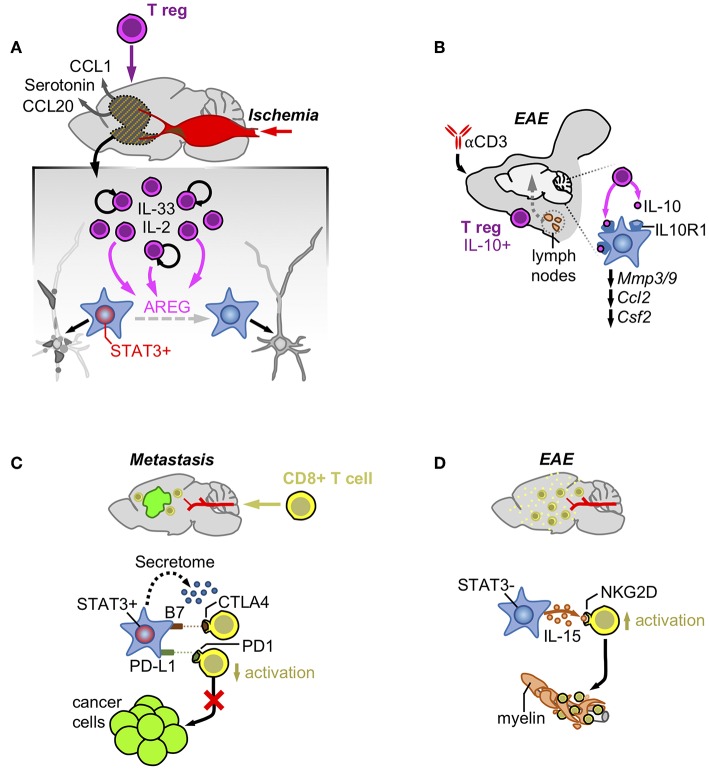
Astrocytes and acquired immunity. **(A)** Ischemia induces the production of CCL1/CCL20/Serotonin that attracts T regulatory cells. Once in the brain, Tregs increase their numbers activated by IL-2 and IL-33 and produce AREG that reduces the neuronal damage by impairing STAT3 activity in astrocytes. **(B)** Treatment with an anti-CD3 blocking antibody intranasally stimulates the production of a subpopulation of T regulatory cells producers of IL-10 at the cervical lymph-nodes. After reaching the brain these cells are responsible for decreasing the expression of genes linked to the pathology by activating the IL-10 receptor in astrocytes. **(C)** Infiltrating CD8+ T cells are exposed to immune checkpoint ligands and an immunosuppressive secretome generated by pSTAT3+ reactive astrocytes that surround established metastasis. **(D)** In contrast, pSTAT3- reactive astrocytes produce IL-15 that binds to NKG2D leading to their increased activation and destruction of myelin.

T regulatory cells have been described to actively modulate astrocyte behavior in ischemia (42). After stroke there is a massive accumulation of T regulatory cells in the brain that promotes neurological recovery. T regulatory cells are initially attracted to the ischemic brain by CCL1 and CCL20 produced by astrocytes and oligodendrocytes and later expanded by the combined action of IL-2 or IL-33 and T cell receptor recognition. Expanded T regs secrete the EGFR ligand amphiregulin (AREG) that decreases the expression of several astrocyte markers associated with potential negative effects on neuronal viability (42) (Figure 2A). In fact, intraventricular administration of AREG reduced neurological dysfunction associated with Treg-depleted-mice (42). Thus, T regulatory cells contribute to the control of brain damage by modulating astrocyte behavior.

Additional evidences of the crosstalk between T regulatory cells and astrocytes exist in EAE. Administration of an anti-CD3 antibody intranasally in this experimental model was shown to activate IL-10-producing T regulatory cells in the cervical lymph nodes (43). These T regulatory cells moved and became enriched in the CNS where they influence reactive astrocytes expressing the IL-10 receptor subunit alfa (*Il10ra*). Activation of IL10R1-dependent signaling in astrocytes decreased gene expression patterns typically associated with different aspects of EAE pathophysiology such as BBB degradation (*Mmp3, Mmp9*), monocyte recruitment (*Ccl2*), and microglial regulation (*Csf2*) (Figure 2B). Interestingly, astrocyte-specific downregulation of *Il10ra* fully impaired the clinical benefit provided by the administration of the anti-CD3 antibody in a pre-clinical model of multiple sclerosis (43).

Primary and secondary brain tumors are infiltrated with CD4+ CD25+ Foxp3+ T regulatory cells (44, 45). In addition, Treg signatures seem to predominate over those related to T-cell function involved in their activation or TCR antigen binding even when measured systemically (46). Consequently, dissecting the biology of the Treg compartment in brain tumors and its cross-talk with other components of the microenvironment including reactive astrocytes might help to develop novel strategies of immunotherapy.

### Cross-Talk Between Astrocytes and CD8+ T Cells

#### Physical Interactions

Cell-to-cell contacts between astrocytes and T cells have been well-documented at the subcellular level (47). Virally infected astrocytes have been imaged *in vivo* when they initiate contacts with T cells in immunized animals (48). These contacts have all the components to be considered an immunological synapse including the central supramolecular activation complex (c-SMAC), composed of the TCR bound to the peptide within the MHC, which is surrounded by the peripheral supramolecular activation complex (p-SMAC), a ring of adhesion molecules including LFA-1 and ICAM-1 (48). The synapse between astrocytes and T cells activates in the later Talin, integrins, and the cytoskeleton that polarizes the T cell to secrete of IFN-γ, perforin, and granzyme-B on the virus-infected astrocyte (48). However, this does not only apply to virally infected astrocytes since the same behavior has been reported in models of multiple sclerosis targeting the gray matter (49) and between transformed glial cells and T cells (50).

However, the presence of immunological synapses between T cells and transformed glial cells do not necessarily correlate with anti-tumor effects, suggesting that astrocytes could negatively modulate T cell activity (51). As part of the neurovascular unit, astrocytes have the important role of blocking potential threats that might get access to a poorly regenerative organ such as the brain. Several mechanisms have proved the efficacy of this natural defense such as the induction of FasL-dependent killing of T cells by reactive astrocytes (52). Interestingly, this mechanism also applies to the elimination of the majority of extravasated metastatic cells that are not adapted to the brain (18). In addition, reactive astrocytes have been shown to inhibit T cells by expressing B7, the ligand of the CTLA-4 checkpoint, whose activation is sufficient to trigger downstream inactivating signals (53). PD-L1 is also present in astrocytes of experimental models of viral encephalitis, where they contribute to limit the function of CD8+ T cells (54), as well as in brain metastases, where the known driver of *Cd274* expression, STAT3, has been shown to be enriched in a subpopulation of these glial cells (19) (Figure 2C). This last finding (the presence or absence of STAT3 in seemingly different astrocyte subpopulations) might underlie the different outcomes after astrocytes and T cells get in contact, emphasizing the importance of dissecting astrocyte heterogeneity in disease.

#### Paracrine Interactions

Astrocyte heterogeneity was initially detected regarding the ability of some of these glial cells to suppress the activation of T cells by unidentified secreted factors (55). More recently, this suppressive activity was linked to the subpopulation of reactive astrocytes activating STAT3 pathway (pSTAT3+) in the context of brain metastasis (19). Although the specific molecular mechanisms mediating these phenomena is still unknown, the secretome of pSTAT3+ reactive atrocytes contained known immunosuppressive molecules and, when evaluated functionally, it impaired the activated state of CD8+ T cells limiting their cytotoxic activity on brain metastatic cells *in vitro* (19) (Figure 2C). The accumulation of reactive astrocytes and CD8+ T cells within the same peri-tumoral area suggests that the paracrine crosstalk between these cell types might play a role *in vivo* (19). In fact, in the context of brain metastasis, where pSTAT3+ reactive astrocytes have been demonstrated to play a critical pro-tumor role, targeting STAT3 in astrocytes and blocking CD8+ T cells simultaneously reverted the decrease in metastasis derived from the loss of function of the transcription factor (19). This finding strongly suggests an important role of pSTAT3+ reactive astrocytes suppressing CD8+ T cells (19).

In contrast, reactive astrocytes in EAE have been shown to produce IL-15, which, upon binding to NKG2D in NK cells and CD8+ T cells (56), stimulates their cytotoxic behavior contributing to increase the damage associated with multiple sclerosis (Figure 2D). Interestingly CD8+ T cells in EAE infiltrate the damaged area leaving the glial cells behind (56), suggesting that the nature of astrocytes might be different to those present in brain metastasis, which retain T cells away from the cancer cells (19).

## Exploiting the Influence of Reactive Astrocytes on the Immune System

The crosstalk between reactive astrocytes and different components of the immune system could have multiple and diverse consequences from neuronal viability to cancer cell proliferation. Thus, in order to target this complex cross-talk therapeutically, it is crucial to understand the role of reactive astrocytes in the specific pathology that is to be challenged. For instance, promoting the crosstalk between Tregs and astrocytes might be a valuable strategy in ischemia and autoimmune disorders but the benefit in the context of cancer is less predictable.

Thus, in primary and secondary tumors the priority is to challenge the survival of cancer cells, which usually hijack mechanisms that are also present in other pathologies and misuse them for their own benefit. There might be associated risks with strategies that look to boost anti-tumor responses by modulating the cross-talk between astrocytes and immune cells such as potential side effects regarding increased direct (due to astrocyte production of neurotoxic molecules) or indirect (due to an overactivation of CD8+ T cells) neuronal damage. Consequently, it is necessary to dissect in great detail the consequences of modulating this cross-talk in pre-clinical models to develop the best strategy for each brain disorder.

Clinical trials have used different strategies that modulate the immune system to treat brain tumors (57–59). Some efforts have reported encouraging results both with primary (58, 60–62) and metastatic tumors (63–65). Nevertheless, response rates remain modest and the question is whether taking into account the specific biology of the brain microenvironment could help to increase them. Given that reactive astrocytes have been proved to influence both branches of the immune system (see above), preclinical studies are needed to define the value of targeting astrocyte-derived local immunosuppression to boost intracranial efficacy of immunotherapies.

In the first place, limited efforts have been devoted to determine the amount of blocking antibodies against immune checkpoints that reach the brain parenchyma compared to extracranial locations (66). Given the presence of the BBB, it is expected that antibody concentrations, if any, will be lower in the brain than elsewhere. The still common argument that the mere presence of a tumor mass involves a disruption of the BBB, which would grant the immediate increase of drug permeability, is far from the reality as reported in exhaustive studies probing that this only affects 10% of fully established metastases (67). Rather than fully disrupted, the BBB seems to be modified into a brain-tumor barrier (BTB), whose biology has just started to be dissected (68).

Thus, if the levels of blocking antibodies reaching the brain parenchyma is a limiting factor, then the anti-tumor effects of such therapeutic approaches will depend on the ability of T cells, activated elsewhere by the action of immune checkpoint inhibitors, to first reach the brain and then get access to tumor cells to apply their cytotoxic activity. Two indirect findings argue in favor of this hypothesis. In experimental brain metastasis models, the presence of systemic disease favors the efficacy of immunotherapy in the brain (69) and, on the contrary, if there is only local disease in the brain, immune cells seem to be sequestered in the bone marrow (70). In other words, immunotherapy based on blocking antibodies solely is not optimized to the particular biology of the brain. Alternatively, the ability of astrocytes to negatively influence immune cells might be exploited to develop novel strategies against brain tumors that could be combined with immune checkpoint blockade.

Reactive astrocytes with activated STAT3 pathway express PD-L1, which could contribute to the local immunosuppressive microenvironment present in brain metastasis (19) (Figure 2B). In fact, cancer cells with glial origin have been shown to induce T cell exhaustion partially due to their expression of PD-L1 (71). In addition, pSTAT3+ reactive astrocytes produce a secretome that impairs the activation state and the cytotoxic phenotype of CD8+ T cells *in vitro* while at the same time promotes the enrichment of pro-tumoral macrophages/microglia that favor the viability of tumor cells (19) (Figures 1C, 2B). In fact, an enriched STAT3 signature brain tumor patients with partial responses to immunotherapy (61). This finding could be interpreted as an active cancer cell-induced mechanism to promote pSTAT3+ reactive astrocytes, which would be responsible for limiting the full potential of anti-tumor T cells thus preventing complete responses. Consequently, BBB-permeable inhibitors targeting STAT3 as well as other inhibitors targeting downstream mechanisms that negatively influence anti-tumor CD8+ T cells and/or impair pro-tumorigenic CD74+ microglia/ macrophages might be explored as a potential combination strategies with immune checkpoint blockade.

Studying the biology of the immune system in the CNS is fundamental to improve therapeutic strategies against brain tumors. The interaction between astrocytes and different branches of the immune system, as extensively proved in other CNS pathologies, suggests a potential avenue to increase the quantity and quality of anti-tumor approaches applied to the brain. The analysis of similar experimental therapeutic approaches across several brain disorders in pre-clinical models might also help to understand the role of astrocytes. For instance, pSTAT3+ reactive astrocytes have been described in brain tumors (19), traumatic injury (72), ischemia (73), neurodegenerative disorders (74, 75) as well as autoimmune diseases (76). However, inhibition of STAT3 in astrocytes is beneficial for some disorders (19, 77–79) while detrimental for others (72).

## Author Contributions

NP and MV conceptualized and wrote the manuscript.

### Conflict of Interest Statement

The authors declare that the research was conducted in the absence of any commercial or financial relationships that could be construed as a potential conflict of interest.
